# Atypical Neurogenesis, Astrogliosis, and Excessive Hilar Interneuron Loss Are Associated with the Development of Post-Traumatic Epilepsy

**DOI:** 10.3390/cells12091248

**Published:** 2023-04-25

**Authors:** Erwin Kristobal Gudenschwager-Basso, Oleksii Shandra, Troy Volanth, Dipan C. Patel, Colin Kelly, Jack L. Browning, Xiaoran Wei, Elizabeth A. Harris, Dzenis Mahmutovic, Alexandra M. Kaloss, Fernanda Guilhaume Correa, Jeremy Decker, Biswajit Maharathi, Stefanie Robel, Harald Sontheimer, Pamela J. VandeVord, Michelle L. Olsen, Michelle H. Theus

**Affiliations:** 1 Department of Biomedical Sciences and Pathobiology, Virginia Tech, Blacksburg, VA 24061, USAeaharris228@vt.edu (E.A.H.);; 2 Department of Cell, Developmental and Integrative Biology, University of Alabama at Birmingham, Birmingham, AL 35233, USA; 3 Department of Biomedical Engineering, Florida International University, Miami, FL 33199, USA; 4 School of Neuroscience, Virginia Tech, Blacksburg, VA 24061, USA; 5 Translational Biology Medicine and Health Graduate Program, Blacksburg, VA 24061, USA; 6 Department of Biomedical Engineering and Mechanics, Blacksburg, VA 24061, USA; 7 Department of Neurology and Rehabilitation, University of Illinois at Chicago, Chicago, IL 60612, USA; 8 Center for Engineered Health, Viginia Tech, Blacksburg, VA 24061, USA

**Keywords:** traumatic brain injury, hippocampus, granular neurons, neuroblasts, seizures, epilepsy, PTE

## Abstract

Background: Traumatic brain injury (TBI) remains a significant risk factor for post-traumatic epilepsy (PTE). The pathophysiological mechanisms underlying the injury-induced epileptogenesis are under investigation. The dentate gyrus—a structure that is highly susceptible to injury—has been implicated in the evolution of seizure development. Methods: Utilizing the murine unilateral focal control cortical impact (CCI) injury, we evaluated seizure onset using 24/7 EEG video analysis at 2–4 months post-injury. Cellular changes in the dentate gyrus and hilus of the hippocampus were quantified by unbiased stereology and Imaris image analysis to evaluate Prox1-positive cell migration, astrocyte branching, and morphology, as well as neuronal loss at four months post-injury. Isolation of region-specific astrocytes and RNA-Seq were performed to determine differential gene expression in animals that developed post-traumatic epilepsy (PTE^+^) vs. those animals that did not (PTE^−^), which may be associated with epileptogenesis. Results: CCI injury resulted in 37% PTE incidence, which increased with injury severity and hippocampal damage. Histological assessments uncovered a significant loss of hilar interneurons that coincided with aberrant migration of Prox1-positive granule cells and reduced astroglial branching in PTE^+^ compared to PTE^−^ mice. We uniquely identified *Cst3* as a PTE^+^-specific gene signature in astrocytes across all brain regions, which showed increased astroglial expression in the PTE^+^ hilus. Conclusions: These findings suggest that epileptogenesis may emerge following TBI due to distinct aberrant cellular remodeling events and key molecular changes in the dentate gyrus of the hippocampus.

## 1. Introduction

Acquired brain injury caused by sudden trauma to the brain disrupts normal function [1,2]. Traumatic brain injury (TBI) remains a significant and growing source of mortality and permanent disability among roughly 2.87 million people yearly in the USA, resulting in profound health and economic burdens [3]. TBI substantially increases the risk of developing comorbidities such as epilepsy, depression, post-traumatic stress disorder, and dementia [4]. Post-traumatic epilepsy (PTE) is a type of acquired epilepsy that represents one of the most common TBI sequelae, affecting up to 50% of individuals after severe TBI and representing up to one-fifth of all acquired epilepsies [5,6,7]. PTE manifests clinically as spontaneous, unprovoked, recurrent seizures, with devastating consequences for patient care, recovery, and overall health [7,8,9].

TBI produces direct and secondary damage to the brain that includes neuronal loss, cerebral blood flow disruption, neuroinflammation, reactive oxidative damage, and blood–brain barrier disruption [10,11,12,13]. These pathophysiological changes may contribute to circuit imbalances that favor excitatory over inhibitory synaptic function [14,15], the formation of spontaneous epileptic foci, and PTE [15,16]. However, the details about the epileptiform process are not well understood from its origin and progression. PTE remains a leading cause of death years after TBI [17,18]. Most patients with PTE have focal with secondarily generalized seizures [19,20,21], but other seizure types are related to hippocampal sclerosis and primary generalized seizures [22,23]. A variety of antiepileptic drugs (AEDs)—including phenytoin [24], levetiracetam [25], lamotrigine, gabapentin, and valproic acid—have been developed to reduce the onset of post-injury seizures, with relative success [26]. While AEDs can be effective in controlling acute or symptomatic seizures (<1 week post-TBI), they do not prevent post-traumatic epileptogenesis, and ~30% of individuals with PTE will develop drug resistance [27,28]. Importantly, these therapies do not target the epileptogenesis process; thus, they do not prevent the late development or progression of PTE [29], and a higher percentage will show AED-related adverse effects that compromise the patient’s quality of life, making AED treatment options ineffective [16,30,31].

Despite rigorous clinical and preclinical research in TBI and PTE, there are no current therapies to prevent the development of PTE, and there is a strong need for better biomarkers and clinical tools that can reliably predict which individuals will develop PTE. Finally, only 7–8% of patients are potential candidates for surgical resection of the epileptogenic foci [32], but surgical complications and difficulties identifying the seizure foci compromise the surgery’s outcomes [16]. The lack of effective treatment highlights the need for robust and reproducible animal models of PTE with spontaneous seizures that are not induced by chemoconvulsants, so as to understand the cellular and molecular changes underlying the development of seizures after TBI [33].

In the present study, we establish that murine focal controlled cortical impact (CCI) injury—an established model of PTE [34,35,36,37,38]—results in PTE onset whose incidence is correlated with the severity of injury. Importantly, we observed significant cellular changes in the hippocampus—namely, aberrant Prox1-positive neuroblast migration, excessive hilar interneuron loss, and altered astrogliosis with divergent transcriptomic signatures that are region-specific. These findings add to the body of work describing hippocampal alterations associated with the development of PTE.

## 2. Materials and Methods

### 2.1. Animals

CD1 male mice at P60–90 from Charles River were housed in an AAALAC-accredited facility with a 12 h light/dark cycle, with food and water ad libitum. For EEG/video recording, the animals were housed individually in 12.5″ × 12.5″ × 15.5″ polycarbonate cages (AAA Plastic Products, Birmingham, AL, USA) with corncob bedding and nesting material. All experiments were conducted in accordance with the NIH Guide for the Care and Use of Laboratory Animals, and with the approval of the Virginia Tech Institutional Animal Care and Use Committee (IACUC; #17-138).

### 2.2. Controlled Cortical Impact (CCI)

The mice were injured as previously described [39,40,41,42,43]. Briefly, ketamine (100 mg/kg), xylazine (10 mg/kg), and buprenorphine SR (0.5 mg/kg) were administered subcutaneously for anesthesia and analgesia before surgery. Hair on the scalp was removed. The mice were then positioned in a stereotaxic frame at 37 °C using a homeothermic blanket system (Harvard apparatus, Lewes, DE, USA). A Φ = 4 mm craniectomy was drilled over the right parietal bone (−2.5 mm A/P and 2.0 mm lateral from bregma), and injury was induced at the center of the craniectomy using a Φ = 3 mm flat tip connected to an eCCI-6.3 device (Custom Design & Fabrication, LLC, Petersburg, VA, USA) at a velocity of 5.0 m/s, with a 250 ms impact duration and a depth of 2.0 mm (*n* = 20) or 2.5 mm (*n* = 23, 15 of which were used for astrocyte isolation). Two severely injured mice (2.5 mm depth) died unexpectedly during the course of the study and were therefore excluded. Kwik-Sil (WPI, Sarasota, FL, USA) was applied to cover the craniectomy, and the incision was closed with 4.0 PDO sutures (AD surgical, Sunnyvale, CA, USA). 

### 2.3. EEG Implantation

Electrode placement was performed 60 days after CCI injury, as previously described [44]. Using a stereotaxic micromotor drill (Stoelting) equipped with a 0.7 mm carbon steel burr drillbit (FST), two holes were drilled through the skull for reference electrodes at coordinates (1.00 ML, 1.00 AP) and (−1.00 ML, −1.00 AP), along with a ground electrode (−1.00 ML, −5.00 AP). Two more holes were drilled partially through the skull, and screws were inserted as anchors. A 0.125 mm diameter platinum–iridium electrode coated in Teflon (Plastics One, Roanoke, VA, USA) was implanted intracranially within 0.5 mm from the surface of the dura. Dental cement (Stoelting, Wooddale, IL, USA) was applied to secure the electrode. Animals were excluded from the experiment if they had profuse hemorrhage during EEG implementation or if they had lost more than 20% of their body weight throughout the study. Sham animals were subjected to craniectomy surgery and electrode implantation. Electrodes were connected to a commutator (Plastics One) using EEG cables (Plastics One), and then to an amplifier (EEG100C, BioPac) with a gain of 5000, a 100 Hz low-pass filter, a 0.5 Hz high-pass filter, and a 500 Hz sampling rate, and they were recorded continuously for two months using BioPac’s AcqKnowledge software, version 4.0.

### 2.4. EEG and Video Analysis

EEG data were analyzed using a MATLAB [45] automated algorithm and manually using BioPac’s AcqKnowledge software (Goleta, CA, USA). We used five criteria to identify seizures. The event duration had to be over 5 s and consist of spikes (20–70 ms), sharp waves (70–200 ms), poly spikes, or slow-wave complexes. We further looked for waveform asymmetry across the x-axis, the evolution of the amplitude and frequency of the spikes over the time course of the event, and postictal suppression of the waveform. When an electrographic seizure was observed, the corresponding video was referenced to determine whether there were any behavioral correlates, and scratching, grooming, or feeding behaviors were observed as sources of artifacts. Mice were considered to be PTE^+^ if they had 2 or more epileptic episodes during the study. Those that did not develop post-traumatic epilepsy were noted as PTE^−^.

### 2.5. Brain Tissue Preparation, Serial Sectioning, and Staining

Tissue handling was performed as previously described [46,47]. Briefly, the mice were euthanatized with isoflurane, followed by transcardial perfusion with 1X PBS, and then 4% paraformaldehyde. Brains were placed in 4% PFA at 4 °C overnight, followed by cryopreservation, embedding in an optimal cutting temperature compound (OCT; Fisher Scientific, Waltham, MA, USA), and storage at −80 °C. Five serial coronal sections of 30 μm, spaced 450 μm apart, were mounted on pre-coated, charged slides using CryoStar Cryostat NX70 (Thermo Fisher Scientific, Highpoint, NS, USA).

### 2.6. Brain Tissue Preparation for Astrocyte Isolation and RNA Extraction

Isolation of cortical and hippocampal astrocytes was performed as described previously [48,49,50]. Briefly, the ipsilateral and contralateral cortices and hippocampi from 15 CCI-injured mice (2.5 mm depth) and 5 shams were microdissected and separated in ice-cold ACSF (120 mM NaCl, 3.0 mM KCl, 2 mM MgCl, 0.2 mM CaCl, 26.2 mM NaHCO_3_, 11.1 mM glucose, 5.0 mM HEPES, 3 mM AP5, 3 mM CNQX) bubbled with 95% oxygen. The tissue was minced, dissociated for 15–30 min using the Worthington Papain Dissociation Kit, and subsequently triturated and filtered through a 70 µM filter until a single-cell suspension was then used to isolate astrocytes utilizing Miltenyi Biotec’s ACSA-2+ MicroBead kit, and then placed on RNAlater (Thermo Fisher Scientific).

### 2.7. RNA Extraction and Sequencing Analysis

RNA isolation was performed on isolated brain-derived astrocytes [48,49]. RNA sequencing was performed by MedGenome. Libraries were prepared using the Takara SMART-Seq V4 ultralow-input RNA kit. Sequencing was performed on a NovaSeq (Illumina, San Diego CA, USA) instrument. Paired-end reads of 2 × 100 bp sequencing runs were performed, with an average of 55 million reads per sample. Bases with quality scores less than 30 and adapters were trimmed from the raw sequencing reads using Trim Galore (v0.6.4). After trimming, only reads with lengths greater than 30 bp were mapped to mm10 by RSEM (v1.2.28) with bowtie2 (v2.4.1), with an average mapping efficiency of 81.5%. The raw counts were used to identify differentially expressed genes by DESeq2 (v1.36.0). Only genes with an average TPM greater than 5 in at least one group, a *p*-value less than 0.05, and at least 1.2-fold change were considered to be differentially expressed genes. All of the differentially expressed genes were used for GO enrichment with the R package clusterProfiler (v4.4.4) and org.Mm.eg.db (v3.15.0). The top 10 most significant biological process (BP) terms were used to generate GO circle plots with the R package GOplot (v1.0.2). GEO accession: #GSE223740.

### 2.8. Immunohistochemistry, Stereo Investigator Analysis, and Lesion Volume

Coronal serial sections were fixed with 10% buffered formalin, washed 3 times in 1 × PBS, and blocked in 2% cold-water fish gelatin (Sigma Aldrich, Inc., St. Louis, MO, USA) with 0.2% triton, before being incubated overnight in block buffer at 4 °C with the following primary antibodies: goat IgG anti-doublecortin (DCX) at 1/200 dilution (Santa Cruz, Santa Cruz, CA, USA; sc-8066) and rabbit IgG anti-Prox-1 at 1/200 dilution (ECM Biosciences, Versailles, KY, USA, #CM4961), with anti-NeuN (Cell signaling technology, Chantilly, VA, USA; #12943S), anti-cFos (Cell signaling technology, #2250), anti-Nestin (Santa Cruz, # sc-33677), or Anti-Cst3 (R&D, # AF1238-SP). After incubation, the slides were washed with PBS 3 times, incubated with secondary antibodies, and mounted in media with DAPI (SouthernBiotech, Birmingham, AL, USA). Images were acquired using a Nikon ECLIPSE Ti2 (Nikon, Melville, NY, USA) inverted confocal microscope with a motorized stage and a Nikon C2 laser system. Five coronal sections were analyzed by a blinded investigator using the Optical Fractionator probe from MBF’s Stereo Investigator software version 2017.03 (MicroBrightField, Williston, VT, USA) and an upright Olympus BX51TRF motorized microscope (Olympus America, Center Valley, PA, USA), as previously described [39,40,42,43]. Contours for the DG and hilus were created, and the optical fractionator’s grid size was set to 150 × 150 mm with a 75 × 75 mm counting frame. The estimated number of cells was then divided by the contoured volume in planimetry and represented as the estimated number of cells per mm^3^. Lesion volume was assessed on five serial coronal sections stained with cresyl violet acetate (Electron Microscopy Sciences, Hatfield, PA, USA) by a blinded investigator using the Cavalieri Estimator from Stereo Investigator (MicroBrightField, Williston, VT, USA) on an upright Olympus BX51TRF motorized microscope (Olympus America, Center Valley, PA, USA), as previously described [41,51,52].

### 2.9. Astrocyte Morphology Analysis

Software-based analysis was used to estimate the coverage, morphology, and branching of the astrocytes in the hilus. Five serial sections were stained for glial fibrillary acidic protein (GFAP), as previously described [43]. Then, 20X z-stack images were used to determine the astrocyte hilar coverage using volumetric analysis, whereas 40X z-stack images were used for morphology and branching analysis. Next, 3D surface reconstructions of GFAP^+^ astrocytes within the hilus were created using Imaris software version 9.6 (Oxford Instruments, Concord, MA, USA) based on absolute threshold intensity, with a smoothing detail of 0.250 µm for morphology analysis, or no smoothing detail for volumetric analysis. For morphology analysis, GFAP-positive astrocytes were separated by using seed points that were approximately equal in size to a typical cell astrocyte soma, calculated in the slicer mode of Imaris. Any surfaces with a voxel size of 10 were excluded from the dataset. Cells that extended outside the region of interest were excluded from the analysis. Morphological comparison of astrocytes in the hilus of PTE^+^, PTE^−^, and sham mice was performed using sphericity, prolation, and oblation. These parameters describe the ellipticity or apparent shape of a spheroid relative to the shape of an astrocyte. Sphericity is computed by the ratio of the surface area of the sphere, which has an equivalent volume to the object over the surface area of the object; this ranges from 0 to 1, where 1 indicates that the object is a perfect sphere [53]. Sphericity can be used to estimate the morphological complexity of the astrocytes. The oblate value refers to how flattened the astrocyte appears, while the prolate value refers to how stretched an astrocyte appears [54].

### 2.10. Sholl Analysis of Astrocytes in Imaris

Following the surface morphology analysis, astrocytes were selected at random to be skeletonized using Imaris’ filament tracer. Filaments were created using the auto path algorithm. Using surface reconstruction as a guide, a region of interest was created around the selected astrocyte. The starting point of the astrocyte was selected by isolating one point at the center of the astrocyte, which could be determined using DAPI, and subsequent seed points were selected by the Imaris quality index with a threshold of +/− 100. Dendrite diameter was selected by Imaris local contrast +/− 0.5 and the nearest point from the distance map algorithm. Following filament creation, 3D Sholl data were extracted from Imaris with a 1 µm increasing shell size. 

### 2.11. Statistical Analysis

Student’s two-tailed *t*-test was used for comparison of the two experimental groups. For three or more groups, multiple comparisons were performed using one-way and two-way ANOVA, with a post hoc test for multiple pairwise examinations. For Sholl analysis, significance was tested using a mixed-effects model with Dunnett’s post hoc analysis for multiple comparisons. Datasets were graphed using GraphPad Prism, version 9 (GraphPad Software, Inc., San Diego, CA, USA). Mean values were reported together with the standard error of the mean (SEM). 

## 3. Results

### 3.1. Murine CCI-Induced PTE Is Associated with Injury Severity and Hippocampal Damage

**Figure 1:**
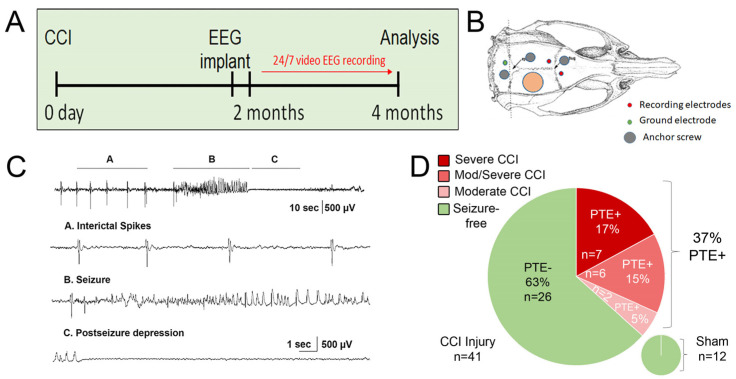
Evaluation of seizure onset following CCI injury: (**A**) Control cortical impact (CCI) injury was induced using either a 2.0 mm or 2.5 mm impactor depth over the right parietal cortex of male adult CD1 mice on day 0. Continuous 24/7 EEG and video recording was performed at 2–4 months post-injury. (**B**) EEG electrode implantation was performed utilizing a bipolar montage composed of two recording electrodes (ipsilateral and contralateral to injury), one ground electrode (green), and two anchor screws to improve retention. (**C**) EEG readings displayed generalized seizures with typical interictal spikes, seizure activity, and post-seizure depression. (**D**) CCI injury was performed on 41 mice (21 at 2.5 mm depth and 20 at 2.0 mm depth) and 12 shams. We observed electrographic and behavioral seizures in 15 CCI-injured and 0 sham-injured mice.

We utilized the controlled cortical impact (CCI) injury model of TBI to monitor and quantify the incidence of PTE in adult male CD1 mice after moderate or severe injury (2 mm or 2.5 mm impact depth, respectively). Two months after the injury, subdural electrodes were implanted. EEG and video recording were performed continuously from 2 to 4 months after injury (Figure 1A), utilizing a bipolar montage with two recording electrodes and one ground electrode (Figure 1B). Electrographic seizures were defined as high-amplitude rhythmic discharges with an amplitude and frequency evolution and a minimum duration of 5 s; epileptiform (interictal) spiking was sometimes present before the onset of the seizure to finalize with postictal depression. Generalized seizures were observed, showing typical interictal spikes, seizure activity, and post-seizure depression (Figure 1C). Using an automated MATLAB algorithm [45], each EEG event was manually confirmed together with a video review. We observed that 37% of all mice tested developed post-traumatic epilepsy (PTE^+^), with a higher incidence seen in mice whose histopathology showed evidence of hippocampal damage or displacement and was categorized as severe (Figure 1D). Animals that did not develop post-traumatic epilepsy were designated as PTE^−^. No seizures were demonstrated in sham-injured mice. Typical epileptic behavior included rapid tremors and muscular spasms from anterior to posterior, lateral recumbency, and rapid tail movements; other mild behaviors were also noted, such as uncoordinated movements or a sudden stop of locomotion, grooming, or eating that was correlated with the duration of EEG activity.

**Figure 2:**
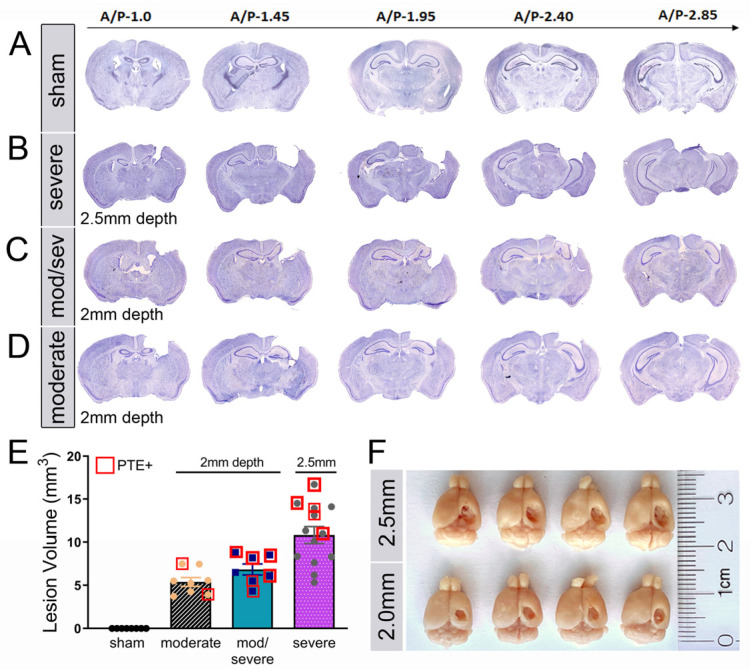
Histological comparison of injury severity in CCI-injured mice: (**A**–**D**) Cresyl violet acetate staining of the serial coronal section of the murine brain from anterior to posterior, encompassing the brain lesion or sham. Based on the quantification of the cavity volume, and hippocampal displacement, there were 4 categories: Sham (**A**), severe (**B**), moderate/severe (**C**), and moderate (**D**) injury based on hippocampal pathology. Centered in each coronal section is a magnified micrograph of the hippocampus. Scale bar = 150 μm. (**E**) Quantified data displaying lesion volume (mm^3^) based on the four categories of histopathology. (**F**) Whole-brain images showing the gross pathology of cavitation induced by unilateral CCI injury between 2.5 mm depth (top panel) and 2.0 mm depth (bottom panel). Total *n* = 15 receiving 2.0 mm depth, *n* = 13 receiving 2.5 mm depth, and *n* = 8 sham, all of which were processed for Nissl staining and stratified accordingly.

Using a 3 mm flat-tip impactor at 2.5 mm depth, we found that, compared to sham mice (Figure 2A,F, Supplementary Figure S1), severely injured mice showed extensive cortical and hippocampal loss with limited posterior intact hippocampal tissue remaining (A/P −2.85) (Figure 2B,F). We also categorized the 2 mm flat-tip impact depth as resulting in two histological subtypes: a moderate/severe phenotype with cortical and partial hippocampal loss (A/P −1.95) (Figure 2C), and a moderate phenotype with cortical tissue loss and intact hippocampi (Figure 2D and Supplementary Figure S1). The quantified lesion volume shows the 2 mm depth moderate injury (5.4 mm^2^ +/− 0.52), with limited PTE incidence compared to moderate/severe (6.8 mm^2^ +/− 0.64) or the 2.5 mm depth severe injury (10.8 mm^2^ +/− 0.52) (Figure 2E). We noted that increased injury severity that included hippocampal damage showed a positive correlation with PTE incidence, i.e., moderate/severe or severe injury (Figure 2E). Only 5% of CCI-injured mice developed PTE whose pathology was consistent with moderate injury, compared to 15% that displayed moderate/severe injury and 17% with severe injury (Figure 1D). Thus, CCI injury that impacts the hippocampus to some degree was correlated with 86% of the PTE^+^ mice in this study. We can conclude that hippocampal function may play a significant role in the development of PTE after focal trauma to the brain.

### 3.2. PTE^+^ Mice Show Aberrant Migration of Prox1-Neuroblasts in the Dentate Gyrus and Hilus

**Figure 3:**
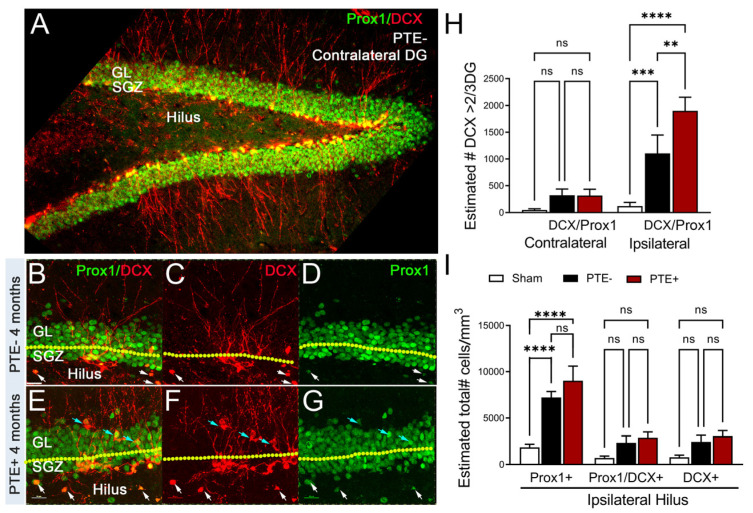
PTE^+^ mice show increased aberrant migration of DCX^+^/Prox1^+^ cells in the dentate gyrus: (**A**) Representative max z-projected confocal image of the contralateral PTE^−^ dentate gyrus (**D**,**G**), showing Prox1 and DCX immunofluorescence. (**B**–**G**) High-magnification max z-projected confocal image at four months in: PTE^−^ (**B**–**D**) and PTE^+^ (**E**–**G**) ipsilateral DG. Prox1 and Prox1/DCX double-labeled cells were observed in the upper two-thirds of the DG (blue arrows) and in the hilus (white arrows). (**H**) Quantified graph showing that PTE^+^ mice display greater increase in the number of Prox1^+^/DCX^+^ cells compared to PTE^−^ mice (*p* = 0.008). (**I**) Quantified graph showing a significant increase in the number of Prox1^+^ cells in the ipsilateral hilus of CCI-injured mice compared to sham mice (*p* < 0.0001). ** *p* < 0.01; *** *p* < 0.001; **** *p* < 0.00001. One-way ANOVA with Bonferroni post hoc correction; *n* = 5–9/group. ns = not significant.

PTE incidence correlates with damage to the hippocampus—a region that is partially responsible for ongoing adult neurogenesis in the subgranular zone (SGZ). The lengthy process of generating new excitatory neurons coincides with the onset of PTE and, therefore, is a likely candidate for aberrant changes in cellular remodeling. We quantified the number of doublecortin (DCX)-positive neuroblasts in the upper two-thirds of the dentate gyrus (DG) and those expressing Prox1—an excitatory granule cell marker [55]—in the hilar region in the contralateral and ipsilateral hemispheres of PTE^−^ and PTE^+^ mice (Figure 3A–D). Under physiological conditions, DCX^+^ neuroblasts are present in the bottom third of the DG. Here, using unbiased stereological analysis, we found a significant increase in the number of aberrant DCX^+^ cells at four months following CCI injury in the ipsilateral > 2/3 DG (*p* = 0.001), which was further increased in PTE^+^ compared to PTE^−^ mice (*p* = 0.009) (Figure 3H).

No significant difference, albeit trending, was found in the estimated numbers of DCX^+^ or double-labeled DCX^+^/Prox1^+^ neuroblasts in the contralateral (data not shown) or ipsilateral hilus (Figure 3I). However, we detected a significant increase in the total number of Prox1^+^ granule cells in the ipsilateral hilus of injured mice compared to sham mice (*p* < 0.0001). The PTE^+^ mice had a slightly higher presence of Prox1^+^ cells, although not significantly different from that of PTE^−^ mice (*p* = 0.23). These findings show the misguidance of Prox1^+^ granule cells in the hilus, which likely differentiated from immature neuroblasts at four months following CCI injury. The occurrence of PTE coincided with disruption to the neurogenic process.

### 3.3. PTE^+^ Mice Display Increased Hilar Expression of c-Fos Alongside an Excessive Loss of Inhibitory Neurons

**Figure 4:**
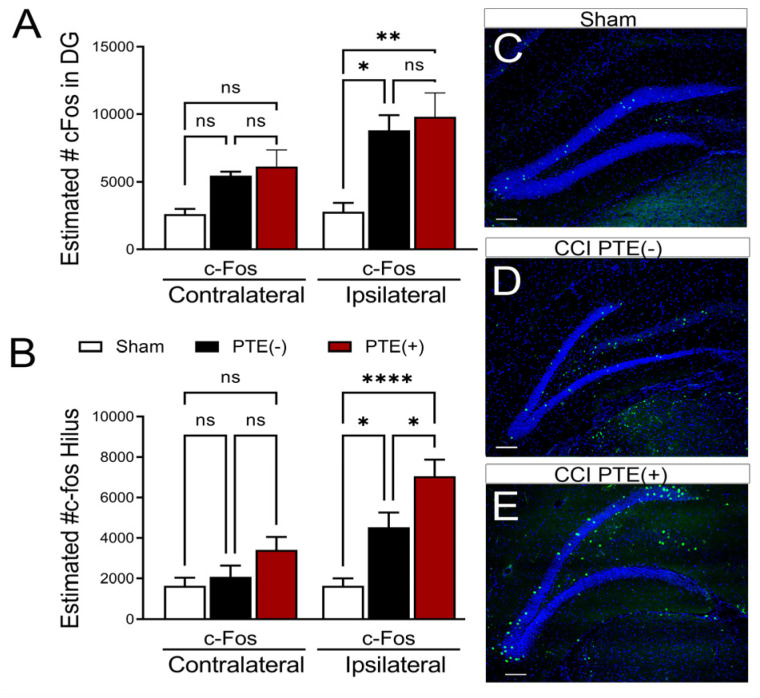
PTE^+^ mice display altered c-Fos in the dentate gyrus: (**A**) Stereological quantification of c-Fos-positive cells (a neuronal activation marker) in the contralateral and ipsilateral DG. Injury induced an increase in the number of c-Fos-expressing neurons relative to sham, regardless of PTE status (PTE^−^ *p* = 0.02; PTE^+^ *p* = 0.002). (**B**) Quantified graph showing no change in contralateral hilar c-Fos; however, PTE^+^ (*p* = 0.0001) and PTE^−^ (*p* = 0.02) are significant compared to sham in the ipsilateral hilus. PTE^+^ mice displayed a greater increase in c-Fos compared to PTE^−^ mice (*p* = 0.03). (**C**–**E**) Representative max z-projection of the dentate gyrus from sham (**C**), PTE^−^ (**D**), and PTE^+^ (**E**) mice stained with DAPI in blue and c-Fos in green. * *p* < 0.05; ** *p* < 0.01; **** *p* < 0.00001. One-way ANOVA with Bonferroni post hoc correction; scale bar = 100 µm; *n* = 5–9/group. ns = not significant. Scale in H = 100 μm.

To evaluate whether neurogenic-mediated changes observed in PTE^+^ mice coincided with altered neuronal activity, we quantified the expression of c-Fos—a trans-synaptic marker of neuronal and astrocyte activity whose expression is increased after seizures [56,57,58,59] in the DG and hilus. We found a trend towards increased numbers of c-Fos^+^ cells in the contralateral DG in PTE^−^ (*p* = 0.36) and PTE^+^ (*p* = 0.18) mice compared to sham mice, although it did not reach statistical significance. We further observed a statistical increase in the ipsilateral (PTE^−^, *p* = 0.029; PTE^+^, *p* = 0.002) c-Fos levels following CCI injury compared to sham levels (Figure 4A). Interestingly, we also found a significant increase in c-Fos^+^ cells in the ipsilateral hilus of PTE^−^ (*p* = 0.028) and PTE^+^ (*p* < 0.0001) mice compared to sham mice (Figure 4B–E). However, the presence of c-Fos^+^ cells was statistically more significant in PTE^+^ mice (Figure 4B,E) compared to PTE^−^ mice (Figure 4B,D) (*p* = 0.03).

**Figure 5:**
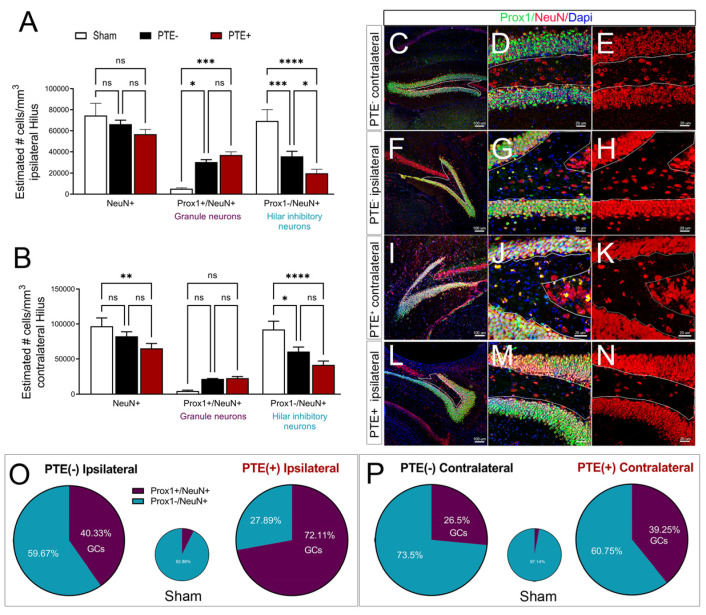
PTE^+^ mice show altered neuronal composition in the hilus: (**A**) Quantified data showing that PTE^+^ mice displayed a trend towards reduced overall numbers of NeuN-positive cells in the ipsilateral hilus, increased numbers of Prox1/NeuN double-labeled granule cells (*p* = 0.005), and reduced numbers of Prox1-negative, NeuN-positive inhibitory neurons (*p* = 0.0001) compared to sham mice. A significant reduction in Prox1^−^/NeuN^+^ cells in PTE^+^ mice was observed compared to PTE^−^ mice (*p* = 0.04). (**B**) Quantified data showing a reduction in the overall numbers of NeuN^+^ cells in the contralateral hilus of PTE^+^ mice compared to sham mice (*p* = 0.004), as well as a reduction in Prox1-/NeuN^+^ inhibitory neurons in CCI-injured PTE^+^ (*p* = 0.0001) and PTE^−^ (*p* = 0.01) mice compared to sham mice. (**C**–**E**) Representative max z-projected confocal images of Prox1 (green) and NeuN (red) in the PTE^−^ contralateral, (**F**–**H**) PTE^−^ ipsilateral, (**I**–**K**) PTE^+^ contralateral, and (**L**–**N**) PTE^+^ ipsilateral hippocampus. (**O**) Proportion of Prox1^+^/NeuN^+^ granule cells/total NeuN vs. Prox1−/NeuN^+^ inhibitory neurons/total NeuN in the ipsilateral hilus of sham, PTE^−^, and PTE^+^ mice. (**P**) Similar proportions displayed for the contralateral hilus. * *p* < 0.05; ** *p* < 0.01; *** *p* < 0.001; **** *p* < 0.00001. One-way ANOVA with Bonferroni post hoc correction; scale bar = 100 µm; sham *n* = 5; PTE^−^ *n* = 5; PTE^+^ *n* = 9. ns = not significant. Scale in low mag = 100 μm and high mag = 20 μm.

To characterize neuronal changes in the hilus that may further delineate c-Fos expression in PTE^+^ and PTE^−^ mice, we quantified the number of NeuN-positive cells (a pan-neuronal marker), Prox1^+^/NeuN^+^ excitatory granule neurons [60], and Prox1^−^/NeuN^+^ hilar inhibitory neurons [61]. Injury reduced the number of NeuN^+^ cells in the PTE^+^ ipsilateral hilus (*p* = 0.06) and was statistically reduced in the PTE^+^ contralateral hilus (*p* = 0.004) compared to sham mice (Figure 5A,C–E). While the number of Prox1/NeuN co-labeled excitatory neurons was increased in both PTE^−^ (*p* = 0.01) and PTE^+^ (*p* = 0.0005) ipsilateral hili compared to shams, no difference was observed between the injury groups (*p* = 0.58). In contrast, both PTE^−^ (*p* = 0.0008) and PTE^+^ (*p* < 0.0001) mice showed reduced numbers of Prox1^−^/NeuN^+^ inhibitory neurons in the ipsilateral and contralateral hilus (*p* = 0.01 PTE^−^; *p* < 0.0001 PTE^+^) compared to sham mice. Interestingly, the loss of inhibitory neurons was more significant in PTE^+^ mice (Figure 5A,I–K) compared to PTE^−^ mice (Figure 5A,F–H) in the ipsilateral hilus (*p* = 0.04). There was also a trend towards reduced numbers in the contralateral PTE^+^ hilus (Figure 5B) (*p* = 0.16).

Finally, the proportions of hilar NeuN^+^ cells that were Prox1^+^ granule cells (Prox1^+^/NeuN^+^ double-labeled cells/total NeuN; GCs) represented only <7% of all neurons in sham conditions. However, CCI injury dramatically elevated this to 40% GCs (Figure 5M, purple), while reducing Prox1-/NeuN^+^ inhibitory neurons to 60% (Figure 5M, blue) of the population in the ipsilateral PTE^−^ hilus. Most notably, PTE^+^ mice showed the opposite trend, with 70% GCs vs. 30% inhibitory neurons. The alterations in GC proportions were also observed in the contralateral hilus between PTE^−^ and PTE^+^ mice, but to a lesser extent. These changes correlated with increased numbers of c-Fos-positive neurons in the hilus of PTE^+^ mice. These data suggest that greater loss of hilar-inhibitory interneurons, complemented by the replacement of excitatory granule cells originating from the neurogenic compartment, may result from or elicit the onset of seizures after TBI. 

### 3.4. Post-Traumatic Epilepsy Alters Hilar Astrogliosis and Astrocyte Morphometric Properties

**Figure 6:**
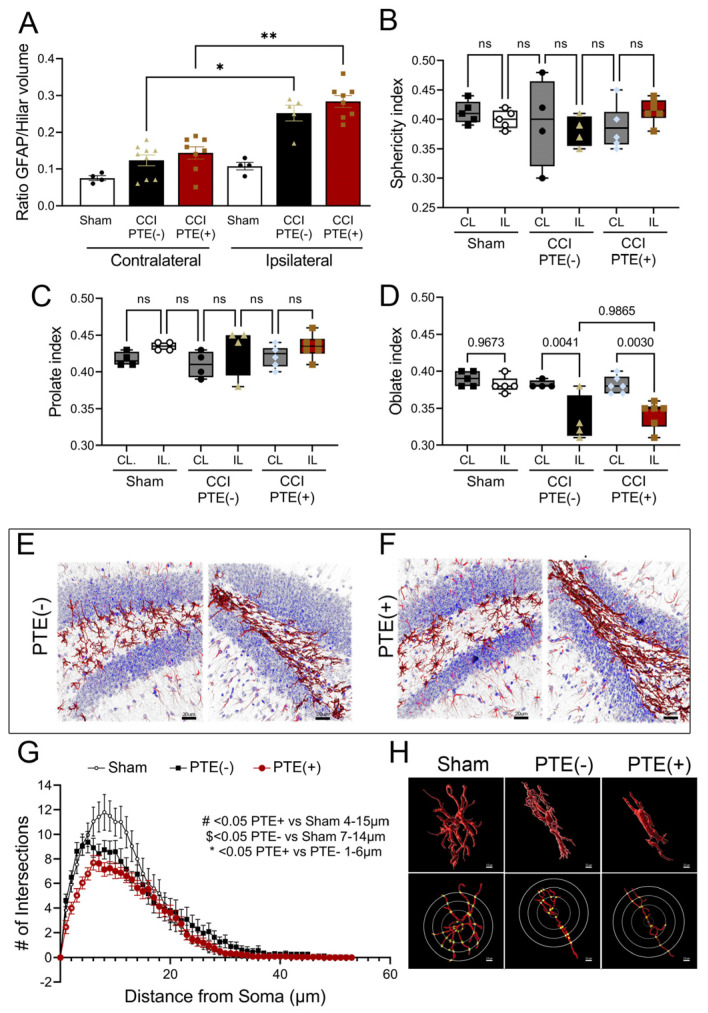
Alterations in hilar astrocyte morphology show distinct changes after CCI injury and reduced branching in PTE^+^ mice: (**A**) Quantified data displaying the ratio of hilar astrocyte coverage. CCI induced an increase in astrocytic coverage in the ipsilateral hilus of PTE^−^ (*p* = 0.03) and PTE^+^ mice (*p* = 0.02) compared to the corresponding contralateral hemisphere. (**B**–**D**) Morphological changes assessed using Imaris software analysis revealed no significant differences in sphericity (**B**) or prolation (**C**). Ipsilateral hilar astrocytes in PTE^+^ (*p* = 0.003) and PTE^−^ (*p* = 0.004) mice showed a reduction in the oblate index (**D**). (**E**,**F**) Representative 3D confocal max z-projected images from PTE^−^ and PTE^+^ hippocampi, respectively. (**G**) Sholl analysis comparing the ipsilateral hemispheres of sham, PTE ^+^, and PTE^−^ animals. PTE^+^ astrocytes showed a reduction in branching within 1 and 6 μm from the cell soma compared to PTE^−^ astrocytes (*p* < 0.05), and within 4 to 15 μm compared to sham astrocytes (*p* < 0.05). Astrocytes from PTE^+^ mice had reduced branching compared to those from sham mice within 2 to 12 μm from the soma (*p* < 0.05). (**H**) A 3D graphical representation of hilar astrocytes in Imaris, displaying an oblong shape and reduced cell processes in injured mice compared to sham mice. * *p* < 0.05; ** *p* < 0.01. One-way ANOVA with Bonferroni post hoc correction; *n* = 4–8/group. ns = not significant. Scale in H = 2.5 μm; E-F = 20 μm.

Astrocytes show structural plasticity in response to synaptic activity and behavior, which contributes to remodeling the surrounding synapses and influences seizure development [62,63,64,65]. Understanding astrocyte morphology is essential to clarify the molecular basis of PTE. We evaluated the % of hilar astrocyte coverage in the hilus of PTE^+^, PTE^−^, and sham mice by calculating the ratio between the GFAP volume and the volume per tissue section. Our results suggest that CCI induces an increase in astrocytic coverage in the ipsilateral hilus of PTE^−^ (*p* = 0.03) and PTE^+^ (*p* = 0.002) mice compared to the corresponding contralateral hemisphere (Figure 6A). Analysis of hilar astrocyte morphology using Imaris was performed to compare the morphological complexity of the astrocytes. The morphological parameters sphericity, prolation, and oblation were calculated in PTE^+^ and PTE^−^ mice. Our results showed altered astrocytic shape in CCI-injured mice. We observed a trend toward increase in the prolation index of astrocytes at the ipsilateral hilus compared to the contralateral hilus in PTE^+^ and PTE^−^ mice (Figure 6C). No difference in sphericity was found (Figure 6B). We detected a decrease in the oblation of astrocytes at the injured ipsilateral hilus compared to contralateral sham, contralateral PTE^−^ (*p* = 0.004), and contralateral PTE^+^ (*p* = 0.003). However, no significant changes in the oblate index were detected between ipsilateral astrocytes of PTE^−^ and PTE^+^ mice, suggesting that these changes could be attributed to the injury rather than to the PTE status. To further characterize the changes in the morphology of astrocytes, we performed a Sholl analysis. Interestingly, we found that PTE^+^ hilar astrocytes showed a reduction in branch complexity at 1 and 6 µm from the cell soma compared to PTE^−^ (*p* < 0.05), and at 4 to 15 µm compared to sham (*p* > 0.05). Additionally, hilar astrocytes from PTE^+^ mice displayed reduced branching compared to those of sham mice at 2 to 12 µm from the soma (*p* < 0.05) (Figure 6G). These findings demonstrate that, as assessed by GFAP immunostaining, astrocytes alter their coverage in the hilus following CCI injury, with a reduction in branching complexity observed in PTE^+^ mice.

### 3.5. Transcriptomic Signature of Forebrain PTE^+^ Astrocytes following CCI Injury

**Figure 7:**
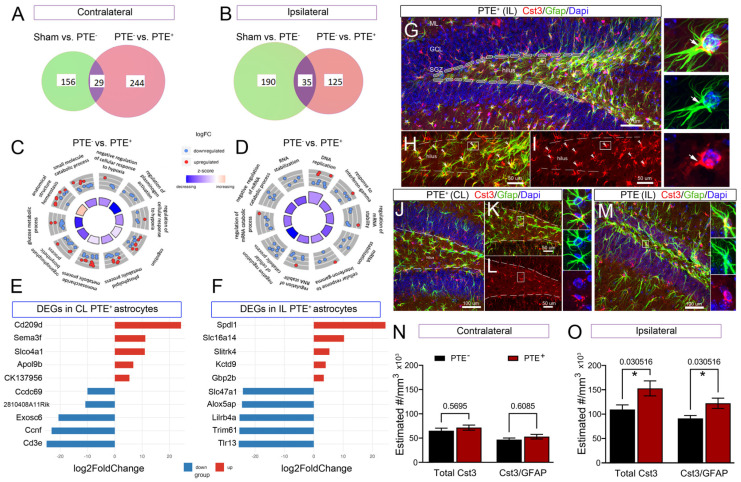
Transcriptomic analysis of hippocampal astrocytes demonstrates key differences in PTE^+^ mice: RNA sequencing was performed on purified astrocytes from the hippocampus. (**A**) Venn diagram DEGs between sham and injured mice in the contralateral hippocampus. (**B**) Venn diagram showing unique DEGs between comparisons in the ipsilateral hippocampus. (**C**,**D**) GO circle plots showing the top 10 gene ontology enrichment terms in PTE^−^ vs. PTE^+^ astrocytes from the hippocampus. These include GO terms associated with cellular response to hypoxia, cognition, and response to interferon-gamma. (**E**) Top 5 up- and downregulated genes in the contralateral and (**F**) ipsilateral PTE^+^ astrocytes compared to PTE^−^ astrocytes. Contralateral: *n* = 5 sham and PTE^+^; *n* = 10 PTE^−^. Ipsilateral: *n* = 5 sham, *n* = 4 PTE^+^, and *n* = 8 PTE^−^. (**G**–**I**) Representative micrographs of the PTE^+^ ipsilateral hilus, (**J**–**L**) PTE^+^ contralateral hilus, and (**M**) PTE^−^ ipsilateral hilus, immunostained against CST3, GFAP, and DAPI. (**H**,**I**) Magnified view of the hilus, showing co-labeling of CST3/GFAP astrocytes (arrowheads). Single-astrocyte micrographs reveal detailed co-localization of stained proteins in the soma of astrocytes. (**N**) Stereological cell counts of CST3^+^ cells and CST3/GFAP double-positives, showing no difference in the contralateral hemisphere of PTE^+^ compared to PTE^−^. (**O**) Ipsilateral hilus showing increased total numbers of CST3 (* *p* = 0.03) and CST3/GFAP (* *p* = 0.03) cells in PTE^+^ mice. CL = contralateral, IL = ipsilateral.

Astrocyte dysfunction has been implicated in the pathophysiology of epilepsy [66,67,68,69], post-traumatic aberrant neurogenesis [70,71], and interneuron degeneration [72]. To gain insight into the molecular pathways associated with astrocyte dysfunction in PTE, we performed RNA-Seq on astrocytes isolated from the ipsilateral and contralateral hippocampus of sham, PTE^+^, and PTE^−^ mice at 4 months following CCI injury. We assessed transcriptomic changes with gene ontology pathway enrichment to detect the most significant DEGs and biological processes (BPs), respectively. Overall, comparing PTE^−^ to PTE^+^, we detected a total of 273 dysregulated protein genes in the contralateral hippocampus: 77 upregulated (top five Log2FC: *Cd209d*, *Sema3f*, *Slo4a1*, and *Apol9b*) and 196 downregulated (top five Log2FC: *Cd3e*, *Ccnf*, *Exosc6*, *Ccdc69* and *2810408A11Rik*) (Figure 7E and Figure 7F; respectively). We identified 160 genes in the ipsilateral hippocampus (Figure 7B): 25 upregulated (top five Log2FC: *Spdl1*, *Slc16a4*, *Slitrk4*, *Kctd9* and *Gbp2b*) and 135 downregulated (top five Log2FC: *Tlr13*, *Trim6l*, *Lilrb4a*, *Alox5ap* and *Slc47a1*) (Figure 7B,F and Supplementary Table S1). Cystatin 3 (*Cst3*) was the only gene altered in PTE^+^ astrocytes in both the contralateral and ipsilateral hippocampus compared to PTE^−^ astrocytes (−0.63, −0.72 Log2FC, respectively; <0.05 *p*-value). To investigate the potential role of Cst3 in hilar astrocytes, we immunodetected CST3 and GFAP astrocytes in coronal sections of the brain (Figure 7G–J), showing intense co-localization of CST3 in GFAP astrocyte soma and, to a lesser intensity, in the astrocyte processes. Stereological cell counts of CST3^+^ cells and CST3/GFAP double-positive cells at the ipsilateral hilus showed increased total CST3 (*p* = 0.03) and CST3/GFAP (*p* = 0.03) in PTE^+^ compared to PTE^−^ (Figure 7M), whereas no significant differences were detected in the contralateral hilus (Figure 7K).

GO circle plots of the top 10 region-specific biological process pathways included those associated with interferon-gamma, response to hypoxia, glucose metabolic processes, and mRNA regulation, among others (Figure 7C,D). Of note, we identified several ‘pan reactive’ markers [73] of astrocytes (including *Lcn2*, *Timp1*, *Cxcl10*, and *Vim*) that were differentially expressed when comparing sham to PTE^−^ in the ipsilateral hippocampus; these differences were not observed in the contralateral hippocampus. These data identify key transcriptomic differences in forebrain astrocytes in mice that develop seizures post-CCI injury. 

## 4. Discussion

Post-traumatic epilepsy (PTE) accounts for 20% of all symptomatic epilepsy and is represented by recurrent generalized or focal seizures with secondary generalization [19,20,21]. Brain contusions and subdural hematomas are the strongest risk factors for seizures, and this increased risk persists for years [5,6,7]. The mechanism by which contusion injury to the brain leads to the subsequent onset of seizures remains under investigation. Using a CCI injury to model cortical contusions, we found that CCI injury of all severity types results in a 37% overall incidence of PTE. In mice that display histopathological evidence of hippocampal damage or displacement, we observed greater occurrence of seizures. These data are consistent with clinical indications that describe the severity of the injury as a significant risk factor for PTE [4,74], as well as with preclinical findings showing that CCI injury results in roughly 40% of male mice developing spontaneous electrographic seizures [37,75]. We further detailed the histopathology of injury severity (including moderate, moderate/severe, and severe) that was associated with the onset of PTE. We demonstrated that hippocampal changes—well known to play a pivotal role in epilepsy [76,77,78]—may be central to the development of PTE. Indeed, previous studies have shown that structural and functional network changes in the dentate gyrus coincide with PTE following CCI injury [37,79,80,81,82,83]. For this study, we utilized a bipolar montage and subcortical electrodes to detect generalized seizures; future studies could utilize multichannel EEG montages with cortical and hippocampal electrodes for localization of seizure origin and follow its progression in the brain. Our findings suggest that cortical and hippocampal damage after TBI is associated with the development of PTE. Future studies targeting the hippocampus using laser ablation or high-frequency irreversible electroporation [84] could aid in establishing a causal role for hippocampal involvement in the development of PTE. 

Our findings show selective loss of Prox1-negative hilar interneurons after CCI injury, and PTE^+^ mice demonstrated a more significant reduction in this cell population than PTE^−^ mice. Prior studies also reported a bilateral selective vulnerability of dentate hilar somatostatin-positive (SST^+^) interneurons following TBI, alongside dentate granule cell hyperexcitability [77], and GAD-67 interneurons were shown to be reduced in the hilus with increasing severity of CCI injury [85]. Here, we found that excessive hilar interneuron loss was associated with mice that developed spontaneous seizures compared to those that did not, suggesting that a threshold of interneuron cell death must be reached, and that targeting this cell population may aid in the prevention of PTE. These data are supported by the fact that hippocampal transplantation of GABAergic progenitors can restore TBI-induced synaptic inhibition and seizure onset [86]. Further studies are needed to discriminate the subtypes of interneurons (e.g., mossy or SST^+^ cells) that are selectively lost in PTE, the mechanisms driving their demise, and conditions associated with TBI. 

We found that PTE^+^ mice showed aberrant migration of Prox1^+^/DCX^+^ immature excitatory granule neuroblasts in the upper two-thirds of the DG of PTE^+^ mice compared to PTE- mice. Importantly, CCI injury resulted in a significant increase in the presence of hilar Prox1^+^ excitatory granule neurons, a fraction of which were Prox1^+^/DCX^+^, indicating that these cells may have matured from SGZ-derived progenitors that were aberrantly migrated [87,88,89] or were granule neurons that dispersed from the DG [90]. It is plausible to assume that this shift from a predominantly Prox1^−^/NeuN^+^-interneuron-populated region (92–97%) to one where Prox1^+^/NeuN^+^ granule neurons reside (72%) may disrupt the excitatory vs. inhibitory balance of the DG circuitry [91,92]. As suggested by our findings of increased numbers of hilar c-Fos-positive cells in PTE^+^ mice, further identification of expression in neurons and astrocytes is needed. Indeed, selective ablation of SGZ neurogenesis can reduce chronic seizure frequency [92], suggesting that mismigration of immature excitatory granule neurons may contribute to aberrant network reorganization. Importantly, this proportional alteration may represent a reliable histological biomarker that could indicate the existence of seizure-susceptible mice.

Interestingly, epileptogenic vulnerable inhibitory interneuron subsets express reelin, whose loss contributes to disrupted neuroblast chain migration in the DG [93]. The interneuron loss may precede and even support aberrant neurogenesis in PTE. Overall, these findings suggest that excessive hilar interneuron loss coincides with excitatory granule cell replacement, partially from the neighboring neurogenic SGZ, which may contribute to the genesis of post-traumatic epilepsy.

Finally, our interrogation of astroglial remodeling in the DG revealed that CCI injury resulted in increased ipsilateral GFAP coverage, indicative of astrogliosis. This increase was not due to the CCI-induced expansion of the GFAP-expressing stem cell population, as demonstrated by the lack of overlap between GFAP and nestin (Supplementary Figure S2). While no difference was observed between PTE^−^ and PTE^+^ hilar area coverage, we did observe significant changes in the oblate index and branching complexity in PTE^+^ hilar astrocytes. These data were consistent with their divergent transcriptomic signatures, which included changes in cystatin 3 (*Cst3*)—a cysteine protease inhibitor, and one of the most highly expressed genes in astrocytes [94,95]—across all brain regions. Glial expression of Cst3 is also associated with abnormal neuroblast migration and neurodegeneration in the epileptic dentate gyrus [71,96], suggesting that alterations in astroglial-specific Cst3 may play a role in network reorganization in the DG of PTE mice. We confirmed CST3 soma localization in astrocytes, and found elevated numbers of CST3^+^ and CST3^+^/GFAP^+^ cells in the ipsilateral hilus of PTE^+^ mice, suggesting that CST3 may be regulate in the morphological changes observed in astrocytes and in the epileptogenesic process after TBI. In addition, we identified a number of genes altered in PTE^+^ astrocytes that are known to be involved in differentiation, migration, and cell morphology (Supplementary Table S2), of which several are associated with epilepsy (i.e., *Eml1* [97], *Map2*, *Map1b* [98], *Sema3f* [99], and *Ptn* [100]). At present, little is known about the role of astrocytes in the health and function of interneurons. Future studies evaluating astrocytic-specific mechanisms in hilar interneuron survival and immature dentate granule cell migration will expand our understanding of epileptogenesis in traumatic brain injury.

## Data Availability

The data presented in this study are available upon request from the corresponding author.

## Supplementary Materials

The following supporting information can be downloaded at: https://www.mdpi.com/article/10.3390/cells12091248/s1, Figure S1: Histological evaluation of injury severity after CCI; Figure S2: Immunofluorescence of GFAP and nestin staining in the DG. Table S1: Top 20 differentially expressed genes from PTE^+^ hippocampal astrocytes vs. PTE^−^. Table S2: Key gene that are differentially expressed in hippocampal PTE^+^ astrocytes.
