# Supplementary material for: Atypical Neurogenesis, Astrogliosis, and Excessive Hilar Interneuron Loss Are Associated with the Development of Post-Traumatic Epilepsy
Source: Cells. 2023 Apr 25;12(9):1248. doi: 10.3390/cells12091248 (PMC10177146; doi:10.3390/cells12091248)
Supplement: Supplementary file 1 [file cells-12-01248-s001.zip › cells-2204286 - Supplementary Figures for XML.pdf]

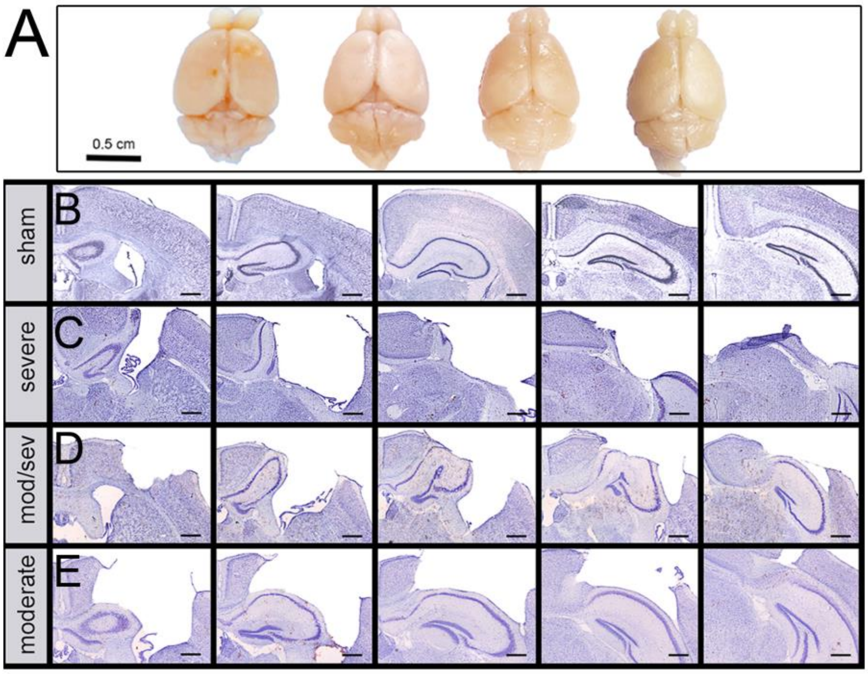

Figure S1. Histological evaluation of injury severity after CCI: (A) Gross image of sham brains before sectioning; (B–E) coronal sections from anterior to posterior (A/P -1 to A/P +2.85), displaying the loss of cortical and hippocampal tissue in severe injury (C), partial loss of hippocampal tissue in moderate/severe injury (D), or unaffected hippocampus in moderate injury (E). The intact cortex and hippocampus can be seen in the sham images (B).

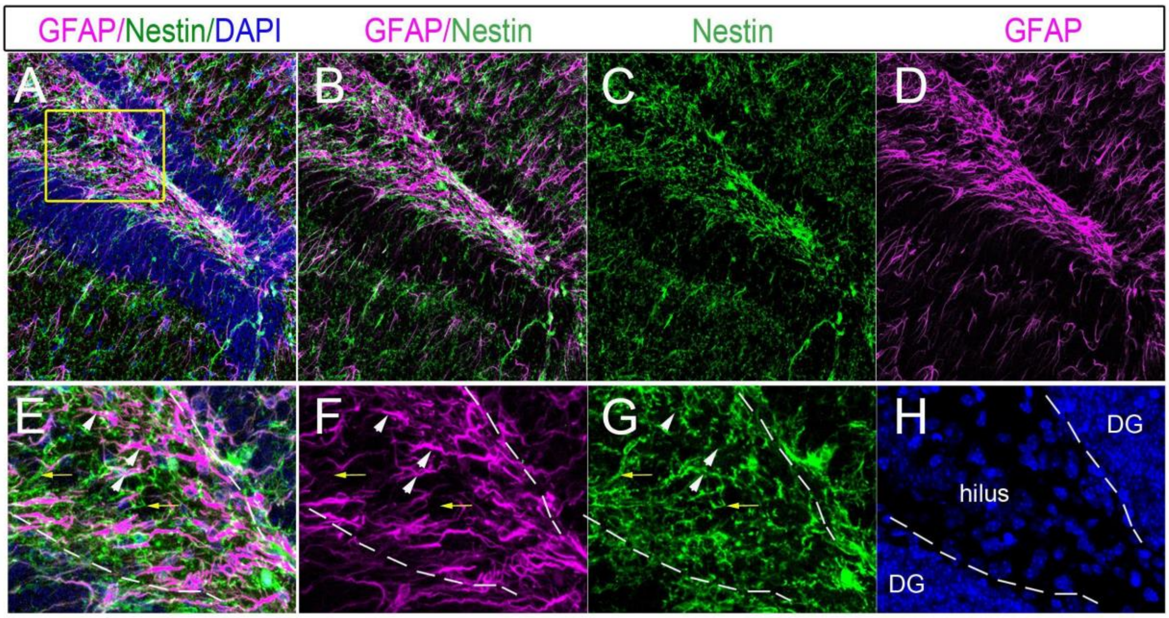

Figure S2. Immunofluorescence of GFAP and nestin staining in the DG: (**A–D**) Max z-projection of 10x confocal images showing GFAP (purple) and nestin (green) staining in the DG at four months post-injury. Merged images

(**A**,**B**) show GFAP and nestin staining co-localized (white) in the SGZ of the DG, but not the hilus. (**E**–**H**) Higher magnification from inset A showing lack of co-localization of hilar astrocytes with the neural stem/progenitor marker nestin. Increased astrocyte coverage in the post-injury hilus is not the result of aberrant migration of the neural stem cell pool, which expresses both GFAP and nestin. DG = dentate gyrus.
