# Supplementary material for: Atypical Neurogenesis, Astrogliosis, and Excessive Hilar Interneuron Loss Are Associated with the Development of Post-Traumatic Epilepsy
Source: Cells. 2023 Apr 25;12(9):1248. doi: 10.3390/cells12091248 (PMC10177146; doi:10.3390/cells12091248)
Supplement: Supplementary file 1 [file cells-12-01248-s001.zip › cells-2204286 - Supplementary Table for XML.pdf]

Table S1. Top 20 differentially expressed genes from PTE^{+} hippocampal astrocytes vs PTE^{-}

Ipsilateral Hippocampus

Genes	log2 Fold Change	P-value	Function
Spdl1	24.4	7.7E-15	Required for the localization of dynein and dynactin to the mitotic kintochore and spindle microtubules.
Slc16a14	10.3	1.0E-03	Transport of monocarboxylates across the plasma membrane.
Elavl3	2.4	5.5E-03	Binds to AU-rich sequences of target mRNAs, including VEGF mRNA. Involved in neuronal differentiation and maintenance.
B630019K06Rik	2.7	1.0E-02	RIKEN cDNA B630019K06 gene.
Slitrk4	5.3	1.2E-02	Involved in synaptogenesis, promotes synapse differentiation and suppresses neurite outgrowth.
Myef2	1.7	1.3E-02	Transcriptional repressor of the myelin basic protein gene (MBP).
Csmd1	2.5	1.9E-02	Cub and sushi domain-containing protein 1; Belongs to the CSMD family.
Zfp202	3.4	2.3E-02	Krab and scan domains-containing zinc finger protein; Zinc finger protein 202.
Zfp334	2.4	2.6E-02	Krab domain-containing zinc finger protein; Zinc finger protein 334.
Jmjd1c	0.8	2.6E-02	Stone demethylase that specifically demethylates 'Lys-9' of histone H3.
Trim61	-25.4	2.1E-15	Apelin receptor early endogenous ligand; acts as a mitogen by promote cell migration
Tlr13	-25.6	2.1E-15	Control host immune response against pathogens through pattern recognition
Lilrb4a	-25.3	3.6E-15	Leukocyte immunoglobulin-like receptor, interferes with TNFRSF5-signaling and NF-kappa-B up- regulation
Alox5ap	-24.5	3.3E-14	Arachidonate 5-lipoxygenase-activating protein; Required for leukotriene biosynthesis.
Slc47a1	-24.2	6.8E-14	Multidrug resistance protein.
Cutal	-10.5	6.8E-09	Periplasmic divalent cation tolerance protein; cutA divalent cation tolerance homolog-like.
Lrrc17	-4.0	1.8E-05	Bone homeostasis. Acts as a negative regulator of RANKL-induced osteoclast precursor differentiation from bone marrow precursors.
Ncf4	-9.8	1.5E-04	Neutrophil cytosol factor 4; Component of the NADPH-oxidase, oxidative burst and ROS regeneration.
Adamts2	-6.5	2.3E-04	Metallopeptidase, disintegrin and metalloproteinase with thrombospondin motifs 2.
Ush1g	-6.9	3.0E-04	Required for normal development and maintenance of cochlear hair cell bundles.

Contralateral hippocampus

Genes	log2 Fold Change	P-value	Function
Sema3f	11.2	1.63E-16	Required for radial and longitudinal organization of myelinated axons and Conduction of nerve impulses in myelinated nerve fibers.
Cd209d	24.2	9.95E-15	Semaphorin-3F; Sema domain, immunoglobulin domain.
Prss35	3.9	1.10E-05	Protease, serine 35; Belongs to the peptidase S1 family.
Slco4a1	11.0	4.54E-04	Mediates the Na(+)-independent transport of organic anions.

Adgrf5	2.1	6.97E-03	Adhesion G protein-coupled receptor. Role in lung surfactant homeostasis
Mprip	0.8	7.15E-03	Required for the regulation of the actin cytoskeleton by RhoA and ROCK1, promotes disassembly of stress fibers in neuronal cells.
Sult5a1	3.1	7.65E-03	Sulfotransferase family 5A, member 1; Belongs to the sulfotransferase 1 family.
Eno2	1.6	7.83E-03	Neurotrophic and neuroprotective properties on a broad spectrum of central nervous system (CNS) neurons, promotes cell survival.
Npy1r	3.0	7.85E-03	Neuropeptide Y receptor type 1; Receptor for neuropeptide Y and peptide YY.
Cd3e	-25.0	5.39E-15	Receptor on T-lymphocyte, role in adaptive immune response during T cell activation by antigen presenting cells.
Ccnf	-23.1	4.38E-13	Substrate recognition component of a SCF, E3 ubiquitin-protein ligase complex, acting as an inhibitor of centrosome reduplication.
Exosc6	-20.6	1.09E-10	MRNA Transport Regulator 3 Homolog. Constitutes one of the subunits of the multisubunit particle called exosome which mediates mRNA degradation.
Cenpa	-6.3	2.19E-08	Role in assembly of kinetochore proteins, mitotic progression and chromosome segregation.
2810408A11Rik	-10.8	5.99E-07	RIKEN cDNA 2810408A11 gene.
Lrrc29	-8.5	6.19E-05	Leucine rich repeat containing 29.
Egr1	-2.6	1.33E-04	Transcriptional regulator inplicated in growth factors, DNA damage, and ischemia. Cell survival, proliferation and cell death.
Ccdc69	-10.0	1.76E-04	Scaffold to regulate the recruitment and assembly of spindle midzone components.
Kif22	-4.2	7.34E-04	Involved in spindle formation and the movements of chromosomes during mitosis and meiosis. Binds to microtubules and to DNA.
Ednra	-1.6	1.02E-03	Receptor for endothelin-1. Mediates its action by association with G proteins that activate a phosphatidylinositol- calcium second messenger system.

Table S2. Key gene that are differentially expressed in hippocampal PTE+ astrocytes

Location	Function	Gene	Fold change (log2)	p-value
Ipsilateral Hippocampus	Proliferation	*Nrtn*	-10.13	0.00
		Eml1	-1.81	0.01
		Prkcz	-1.74	0.02
	Migration	*Elavl3*	2.40	0.01
		Trim61	-25.36	0.00
		Mien1	-1.24	0.03
		Gm13306	-1.99	0.04
	Cell morphology	*Slitrk4*	5.33	0.01
		Sept3	-0.81	0.02
Contralateral Hippocampus	Proliferation	*Egr1*	-2.63	0.00
		Fos	-2.22	0.00
		Lsamp	-0.37	0.00
		Mdk	-0.60	0.01
		Ptn	-0.36	0.02
		Eno2	1.56	0.01
	Migration	*Map2*	0.71	0.02
		Unc5a	3.00	0.04
	Cell morphology	*Sema3f*	11.17	0.00
		Mprip	0.77	0.01
		Map2	0.71	0.02
		Map1b	1.17	0.05
